# Global warming hiatus contributed to the increased occurrence of intense tropical cyclones in the coastal regions along East Asia

**DOI:** 10.1038/s41598-018-24402-2

**Published:** 2018-04-16

**Authors:** Jiuwei Zhao, Ruifen Zhan, Yuqing Wang

**Affiliations:** 1 Pacific Typhoon Research Center, Key Laboratory of Meteorological Disaster of Ministry of Education (KLME), Nanjing University of Information Science and Technology, Nanjing, China; 2 International Pacific Research Center and Department of Atmospheric Sciences, School of Ocean and Earth Science and Technology, University of Hawaii at Manoa, Honolulu, Hawaii USA; 3 Shanghai Typhoon Institute of China Meteorological Administration, Shanghai, China

## Abstract

The recent global warming hiatus (GWH) was characterized by a La Niña–like cooling in the tropical Eastern Pacific accompanied with the Indian Ocean and the tropical Atlantic Ocean warming. Here we show that the recent GWH contributed significantly to the increased occurrence of intense tropical cyclones in the coastal regions along East Asia since 1998. The GWH associated sea surface temperature anomalies triggered a pair of anomalous cyclonic and anticyclonic circulations and equatorial easterly anomalies over the Northwest Pacific, which favored TC genesis and intensification over the western Northwest Pacific but suppressed TC genesis and intensification over the southeastern Northwest Pacific due to increased vertical wind shear and anticyclonic circulation anomalies. Results from atmospheric general circulation model experiments demonstrate that the Pacific La Niña–like cooling dominated the Indian Ocean and the tropical Atlantic Ocean warming in contributing to the observed GWH-related anomalous atmospheric circulation over the Northwest Pacific.

## Introduction

Tropical cyclones (TCs) can cause enormously devastating losses of human life and property damages, especially for coastal areas, including China, Korea, and Japan over East Asia^1–3^. With the rapid increase in populations and economic growth, coastal countries in East Asia are likely to face greater risk during intense TC (ITC) events. Therefore, regional change in ITC activity in the coastal regions along East Asia over the western Northwest Pacific is a critical scientific and socioeconomic issue.

A large increasing trend in the number and proportion of category 4–5 TCs has been observed over the main TC basins in recent decades^1,4–9^. This increase has often been linked to global warming^7,10–13^. Global warming not only induces the increase in sea surface temperature (SST) but also enhances the upper-ocean stratification due to the reduction of surface salinity by the upper-ocean freshening^11^. The latter in turn reduces the TC-induced vertical mixing and sea surface cooling and favors the intensification of ITCs^10,11,14,15^.

Recent studies have also reported a significant westward shift of the prevailing TC tracks over the Northwest Pacific with an increase in landfalling ITCs in recent three decades^3,16^. This westward shift of prevailing TC tracks has been attributed to the expansion of subtropical high over the Northwest Pacific and local ocean surface warming^3,17,18^. Since both the increase in landfalling ITCs and the westward shift in TC tracks over the Northwest Pacific imply the increasing threat by more ITCs to the coastal regions of the East Asian countries, it is important to further explore the regional change in ITC occurrences and understand possible mechanisms that contributed to the related regional change based on a perspective of global SST anomalies.

The global surface warming over the 20th century was found to slow down during 1998–2013. This phenomenon was referred to as the global warming hiatus (GWH) and has been discussed in numerous studies^19–26^. The GWH was shown to be characterized by a La Niña–like cooling in the tropical eastern Pacific (EP) accompanied with the Indian Ocean (IO) and the tropical Atlantic Ocean (AO) warming. Previous studies have proposed that the GWH could be triggered by the internal variability of the coupled ocean-atmospheric system, such as the Pacific Decadal Oscillation, or the external natural forcing, such as volcanic eruption and aerosols, or both^19,20,24,27,28^. Regardless of its triggering mechanisms, the GWH has been demonstrated to have imposed important impact on the North American climate and the Asian monsoon variability^29,30^.

Here we will report a new finding that the recent GWH contributed significantly to the regional changes of ITC activity over the Northwest Pacific, in particular the increase in the coastal regions along East Asia. This has been demonstrated by the Singular Value Decomposition (SVD) analysis, which allows us to examine the relationships between the regional variability of TCs over the Northwest Pacific and climate variability on global scales.

## Results

**Figure 1:**
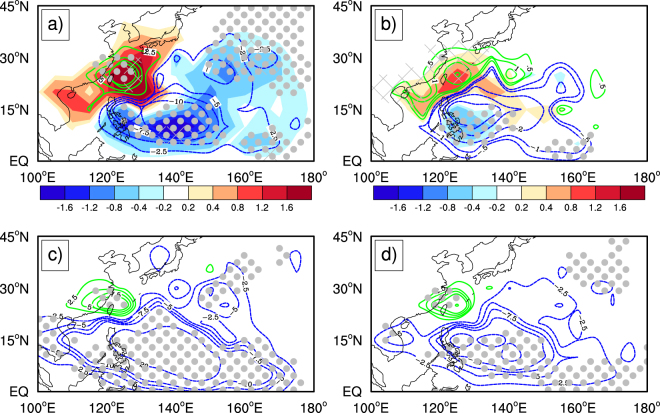
Trends and composite differences in the observed ITCTs. (**a**) Trends in ITCTs (shades; counts per decade) during 1980–2015 and composite differences in the observed ITCTs between the hiatus (1998–2015) and pre-hiatus (1980–1997) periods (contour; counts per decade); (**b**) same as (**a**) but calculated for the points where TCs reached ITC only (with the SMWS larger than 96 knots); (**c**) composite differences in ITCTs between the hiatus (1998–2009) and pre-hiatus (1982–1997) periods using the ADT-HURSAT data; and (**d**) same as (**c**) but using the JTWC TC best-track data. Areas where composite differences are statistically significant above the 90% confidence level are shown in grey dots based on the Mann-Whitney U test. The slashes in (**a**) and (**b**) represent areas where the trend is statistically significant above the 90% confidence level based on Student’s t test. This figure was generated by the NCAR Command Language (Version 6.4.0) [Software]. (2017). Boulder, Colorado: UCAR/NCAR/CISL/TDD.

Figure 1a shows the spatial distributions of the linear trend in ITC tracks (ITCTs) over the Northwest Pacific during 1980–2015 (shades) and the composite difference in ITCTs between the hiatus (1998–2015) and pre-hiatus (1980–1997) periods (contours). Here the ITCTs were defined as the frequency of ITC occurrence in each 5° × 5° grid box. There is a significant increasing trend in ITCTs along the East Asian coasts over the western Northwest Pacific, west of 135°E, and a decreasing trend over the southeastern Northwest Pacific. This northwest-southeast dipole trend pattern also appears in composite difference in ITCTs between the hiatus and pre-hiatus periods. This consistency suggests that the GWH might contribute to the observed trends in ITCTs over the Northwest Pacific. The pattern in ITCTs only including the points where TCs reached ITCs (Fig. 1b) is quite similar to that in ITCTs for TCs reaching ITCs in their lifetimes shown in Fig. 1a. Since the sample size for the latter is much larger than that for the former, we will focus on the analyses for the latter below. To confirm the reliability of the trend analysis based on the JTWC TC best-track dataset, we compared the trends in ITCTs based on the ADT-HURSAT dataset and on the JTWC TC best-track dataset during 1982–2009 in Fig. 1c and d, respectively. Overall, the trend patterns from the two datasets are very similar to each other, suggesting that the analysis based on the JTWC TC best-track data is reliable and independent of the TC best-track dataset used.

**Figure 2:**
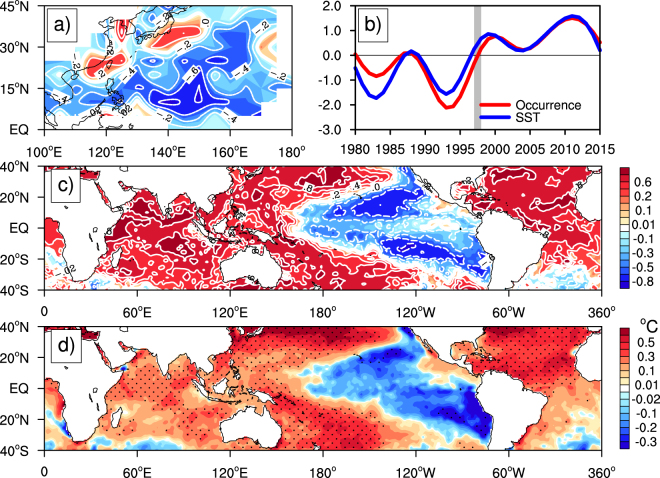
Spatial and temporal structures of the first SVD mode between ITCTs over the Northwest Pacific and global SST anomalies. (**a**) Singular eigenvector of the normalized ITCTs, (**c**) singular eigenvector of the normalized SST anomalies, (**b**) principal components (PCs) of the ITCTs and SST anomalies in non-dimensional units; and (**d**) composite differences in SST anomalies (°C) between the hiatus (1998−2015) and pre-hiatus (1980–1997) periods. The correlation coefficient between the two PCs in (**b**) is 0.91, which is significant at 99% confidence level based on the Student's *t* test after adjusting the effective degrees of freedom. Areas with the confidence level above 90% are shown by dots in (**d**). This figure was generated by the NCAR Command Language (Version 6.4.0) [Software]. (2017). Boulder, Colorado: UCAR/NCAR/CISL/TDD.

To demonstrate the contribution by the GWH to the observed regional trends in ITCTs over the Northwest Pacific, we did the SVD analysis for the frequency of ITC occurrence over the Northwest Pacific and the global SSTAs between 40°S and 40°N averaged in the typhoon seasons during 1980–2015. As mentioned in the Methods section, the interannual signals in the ITCT and SST data were removed before the SVD analysis. The first SVD mode shows interdecadal increasing trends in ITCTs and global SSTAs (Fig. 2b). The squared covariance percentage explained by this mode reaches 49%, significant at the 90% confidence level based on Monte Carlo significance test^31^. The time series of the two first SVD modes are predominantly negative before 1998 and positive since 1998 (Fig. 2b). Their correlation coefficient is as high as 0.91, significant at the 99% confidence level by Student’s *t-*test after adjusting the effective degrees of freedom^32^. The results thus indicate that the first SVD mode of ITCT trends (Fig. 2a) is well coupled with that of SSTAs characterized by a uniform SST warming trend over the tropics except for an SST cooling trend appeared over the eastern Pacific (Fig. 2c). The similarity between Fig. 1a and 2a and that between Fig. 2c and 2d demonstrate that the pattern in the linear trends in ITCTs shown in Fig. 1a is closely tied to the recent GWH characterized by SSTA pattern shown in Fig. 2b. That is why we can divide the whole studied period into two sub-periods: the pre-hiatus (1980–1997) and the hiatus periods (1998–2015) based on the time series of SSTA derived from the first SVD mode (Fig. 2b). This is also consistent with previous studies^30,33^.

**Figure 3:**
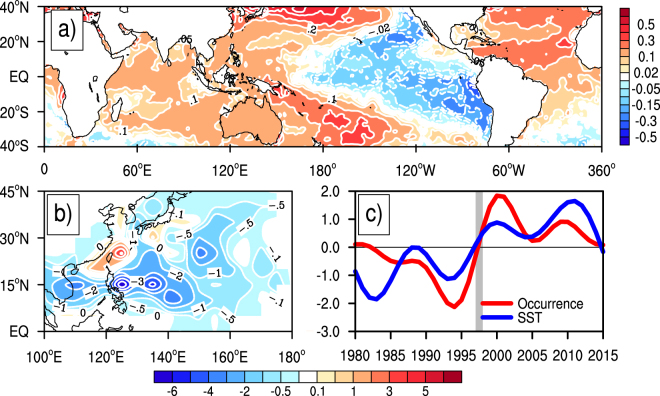
Spatial and temporal structures of the first EOF modes of global SST anomalies and ITCTs over the Northwest Pacific. (**a**,**b**) The spatial patterns for the first EOF modes of (**a**) the global SST anomalies and (**b**) ITCTs over the Northwest Pacific with the corresponding interannual variabilities removed, and (**c**) the time series of their principal components with red for ITCTs and blue for SST. This figure was generated by the NCAR Command Language (Version 6.4.0) [Software]. (2017). Boulder, Colorado: UCAR/NCAR/CISL/TDD.

For a comparison, we also performed the Empirical Orthogonal Function (EOF) analyses of the global SST anomalies and the ITCTs over the Northwest Pacific, respectively. In general, the first EOF modes (Fig. 3) are very similar to the coupled mode based on the SVD analyses shown in Fig. 2. The spatial pattern for the first EOF mode of the global SST anomalies also shows the recent GWH mode, characterized by a La Niña-like SST cooling pattern in the Pacific region accompanied with the IO and AO warming (Fig. 3a). The first EOF mode of the ITCTs over the Northwest Pacific displays an increased ITC occurrence over the East Asian coastal region and a decreased ITC occurrence over the southeastern Northwest Pacific in the hiatus period (Fig. 3b). The time series of the two first EOF modes show negative anomalies before 1998 and positive anomalies since 1998 (Fig. 3c), with contributions of 45.6% and 38.6%, respectively, to the corresponding total variances. The similarity between the EOF and SVD analyses strongly suggests that the results based on the SVD analyses above are robust.

**Figure 4:**
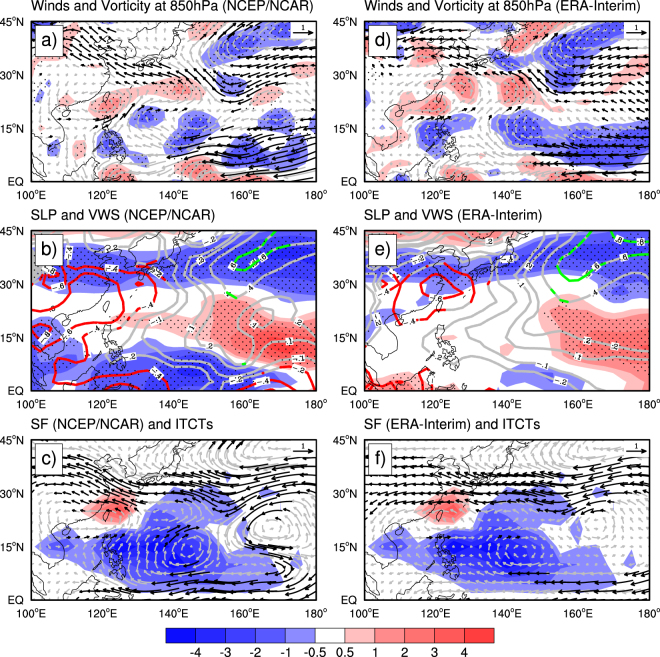
Influence of global hiatus on the large-scale environments over the Northwest Pacific. Composite differences in (**a**,**d**) 850-hPa wind (vectors in m s^−1^) and 850-hPa vorticity (shades in 10^−6^ s^−1^), (**b**,**e**) SLP (contours in hPa) and VWS between 200 and 850 hPa (shades in m s^−1^), and (**c**,**f**) mass-weighted mean winds between 300 and 850 hPa (SF, vectors in m s^−1^) over the Northwest Pacific between the hiatus (1998–2015) and pre-hiatus (1980–1997) periods based on (**a**–**c**) the NCEP/NCAR and (**d**–**f**) the ERA-Interim reanalysis data. Shades in (**c**) and (**f**) indicate the differences in ITC occurrence in the two periods as in Fig. 1a but divided by factor of 5 for sharing the same color bar. All difference fields were averaged in JASON. Black vectors, red and green lines, and dots represent areas where the differences are statistically significant at the 90% confidence level using Mann-Whitney U test. This figure was generated by the NCAR Command Language (Version 6.4.0) [Software]. (2017). Boulder, Colorado: UCAR/NCAR/CISL/TDD.

To understand how the GWH affected the regional changes in the ITCTs, we examined the changes in the large-scale atmospheric circulation over the Northwest Pacific in response to the GWH. Figure 4 shows the composite differences in winds and relative vorticity at 850 hPa, sea level pressure (SLP), vertical wind shear (VWS) between 200 and 850 hPa, and the mass-weighted mean winds (or steering flow) from 850 to 300 hPa between the hiatus and pre-hiatus periods based on both the NCEP/NCAR reanalysis (Fig. 4a–c) and the ERA-interim reanalysis (Fig. 4d–f). We can see that the overall patterns of these changes from the two independent reanalysis datasets are highly consistent. During the hiatus period, the equatorial easterly anomalies dominate the tropical western Pacific, with an anomalous cyclonic circulation with positive vorticity anomalies and reduced SLP north of 20°N and extending westward from 145°E to East Asia, and an anomalous anticyclonic circulation with negative vorticity anomalies and increased VWS dominates the southeastern Northwest Pacific between 120°E and 160°E. The former is favorable for TC genesis and intensification over the western Northwest Pacific, while the latter greatly suppresses TC genesis and intensification locally^34,35^. Furthermore, the southeasterly steering flow north of 20°N over the western Northwest Pacific (Fig. 4c and f) favored more TCs to head to the coastal regions of East Asian countries. These explain why the ITCTs significantly increased over the western and decreased over the southeastern Northwest Pacific during the hiatus period.

Changes in the atmospheric circulation over the Northwest Pacific are primarily driven by the tropical oceanic conditions^30,33^. For example, the intensification of the Walker circulation was closely related to the La Niña-like SSTA pattern, which has been considered as one of the main feature of the GWH^19,22–26^. The intensified Walker circulation induced low-level easterly anomalies, suppressing TC genesis and frequency of occurrence over the southeastern Northwest Pacific. In sharp contrast, the western Northwest Pacific was controlled by local SST warming and an anomalous cyclonic circulation^3,34,35^, which was favorable for TC intensification. In addition to the La Niña-like SSTA pattern, the composite difference of SSTA in the two periods also shows positive SSTAs over the IO and AO (Fig. 2d) as also shown in the first SVD SST mode (Fig. 2c). Some previous studies have proposed that the IO and AO SSTAs can also play important roles in the Walker circulation strengthening and changes in the atmospheric circulation over the Northwest Pacific and thus the TC activity over the Northwest Pacific, in particular on interannual time scales^26,30,36–40^. To see the relative contributions of the GWH-related SSTAs in different ocean basins (namely the Pacific Ocean, the IO, and the AO) to the long-term changes in atmospheric circulation over the Northwest Pacific identified based on reanalysis data discussed above, we conducted a series of sensitivity experiments as summarized in Table 1.

**Table 1:** Experimental Design of Atmospheric General Circulation Model (AGCM) Simulations.

Name	Region	SST
Control run (CTRL)	Global	Observed climatological monthly mean SST during 1980–2015.
Hiatus run (HTS)	60°S–60°N; 0°–360°	Identical to CTRL except that the individual monthly mean SST anomalies between the hiatus (1998–2015) and pre-hiatus (1980–1997) periods were added to the belt region.
Pacific Ocean (PO)	60°S–60°N; 110°E–60°W	Identical to CTRL except that the individual monthly mean SST anomalies between the hiatus and pre-hiatus periods were added to the Pacific Ocean.
Indian Ocean (IO)	60°S–60°N; 0°–110E°	Identical to CTRL except that the individual monthly mean SST anomalies between the hiatus and pre-hiatus periods were added to the Indian Ocean.
Atlantic Ocean (AO)	60°S–60°N; 60°W–0°	Identical to CTRL except that the individual monthly mean SST anomalies between the hiatus and pre-hiatus periods were added to the Atlantic Ocean.

**Figure 5:**
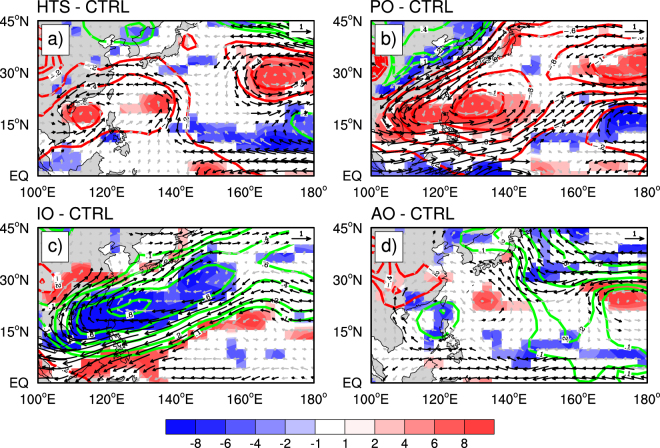
The relative contributions of the GWH-related SSTAs in different ocean basins. Differences in the simulated SLP (green and red contours, unit: hPa), 850 hPa relative vorticity (blue and red shades, unit: 2 × 10^−7^ s^−1^) and winds (grey and black vectors, unit: 0.8 m s^−1^) between (**a**) the HTS run, (**b**) the Pacific Ocean (PO) run, (**c**) the Indian Ocean (IO) run, (**d**) the Atlantic Ocean (AO) run, and the control (CTRL) run. All difference fields were averaged in JASON. This figure was generated by the NCAR Command Language (Version 6.4.0) [Software]. (2017). Boulder, Colorado: UCAR/NCAR/CISL/TDD.

Figure 5 shows the difference fields in the simulated SLP, 850 hPa relative vorticity and winds between each of the four sensitivity experiments and the control run (CTRL run). The model quantitatively reproduced the observed decadal anomalies in low-level winds, relative vorticity and SLP over the Northwest Pacific as seen in the hiatus run (HTS run) minus the CTRL run (Fig. 5a). An anomalous cyclonic circulation with positive vorticity anomalies occupies the area from East Asia to the Northwest Pacific west of 140°E, accompanied with equatorial easterly anomalies over the southeastern Northwest Pacific, although the simulated anticyclonic circulation to the east of the Philippines is slightly weaker and more eastward than that observed. This demonstrates that the model reproduced the main features in the large-scale atmospheric circulation changes in response to the recent GWH. The low-level circulation difference between the PO run and the CTRL run (Fig. 5b) is characterized by a strong anomalous cyclonic circulation with positive vorticity anomalies over the Northwest Pacific and relatively weak equatorial easterly anomalies over the eastern part of tropical western Pacific, similar to those shown in Fig. 5a except that the simulated anomalous cyclonic circulation is somewhat stronger. This suggests that the Pacific SSTAs with the warming in the western Pacific and cooling in the eastern Pacific contributed largely to the observed circulation changes induced by the GWH. The low-level circulation differences between the IO run and the CTRL run shows a strong anomalous anticyclonic circulation accompanied by positive SLP anomalies over the Northwest Pacific (Fig. 5c), similar to the atmospheric response to the IO warming found in previous studies^39–41^ but is different from those shown in Fig. 5a. This suggests that the IO warming contributed negatively to the ITCT trend induced by the SSTAs over the Pacific Ocean as shown in Fig. 5b. Finally, the low-level circulation differences between the AO run and the CTRL run are considerably weak except for the equatorial easterly anomalies (Fig. 5d). This confirms the hypothesis of previous study^30^, namely contribution by the Atlantic warming to the regional trends in ITCTs over the Northwest Pacific is likely to be secondary. These results demonstrate that the GWH, especially its SSTA pattern in the Pacific, played predominant roles in the strengthened ITC activity in the coastal regions along East Asia over the western Northwest Pacific.

## Conclusions and Discussion

We have investigated the long-term trend in ITC tracks over the Northwest Pacific and its relationship with global SST changes based on observations and a set of general circulation model experiments. We found that there is an increasing trend in ITCTs in the coastal regions along East Asia over the western Northwest Pacific since 1998, posing increasing coastal risk to East Asian countries, while a downward trend over the most of the southeastern Northwest Pacific. Our results demonstrate that the recent La Niña-like SST anomalies, which is often dubbed the GWH in the period 1998–2013, is responsible for the significant trends in ITCTs. The recent GWH strengthened an anomalous cyclonic circulation over the western Northwest Pacific, which provided favorable conditions for TC genesis and intensification, intensifying ITC activity along East Asia. The GWH also induced equatorial easterly anomalies, an anomalous anticyclonic circulation with negative vorticity anomalies, and increased VWS in the southeastern quadrant of the Northwest Pacific, greatly suppressing TC genesis and intensification and thus leading to the decreasing trend in ITCTs over the southeastern Northwest Pacific. Results from numerical experiments further demonstrate that the Pacific La Niña-like SST cooling contributed predominantly to the observed ITCT trend, and contribution by the AO warming were secondary, while the IO warming played an opposite role.

The strengthened equatorial easterly over the western Pacific in the recent hiatus period is associated with the La Niña-like SST pattern addressed in previous studies^19,22,38^. The IO and AO SSTAs are also shown to contribute to the strengthened Walker circulation^30,38^. As mentioned in section 1, some external forcings, such as natural volcanic eruption and aerosol forcings, might also have played some roles in intensifying the Walker circulation^20,28^, but these have not been considered in this study.

Furthermore, the coupled SSTA pattern shows a similarity to the mega-ENSO pattern over the Pacific as shown in aforementioned studies^35,42^. The GWH exhibits the negative phase of mega-ENSO. This suggests that there exists a coupled mode in the Pacific with a time scale longer than the decadal/interdecadal time scales. This possibility is hard to be detected at present because the relatively reliable TC intensity data are not long enough for such an analysis. Nevertheless, it should be noted that although some previous studies attributed the increasing trends in ITCs to global warming^3^, care needs to be taken when a projection is made for the future since the global warming often displayed a permanent negative phase of mega-ENSO or a negative phase of the coupled trends identified in this study. Finally, the relative importance of the local SST warming over the western Northwest Pacific and the basin scale or global scale SSTA modes needs to be addressed in future studies.

## Methods

The TC best-track data during 1980–2015 over the Northwest Pacific obtained from the Joint Typhoon Warning Center (JTWC) were used in this study, including the location of each TC center and 1-minute averaged surface maximum sustained wind (SMSW) at 6-h intervals. An ITC was defined as a TC with the SMSW larger than 96 knots (namely, categories 3, 4 and 5 TCs) according to the Saffir-Simpson scale. The ITC tracks (ITCTs) were defined as the frequency of ITC occurrence in each 5° × 5° grid box. The frequency of occurrence meant how often TCs affected a specific grid box. Here, to minimize the subjectivity in the identification of weak systems, the ITCTs only considered the locations with SMSW ≥ 34 knots (tropical storms) in the whole lifetimes of ITCs. To address the uncertainties in the trend analysis using the TC best track data^3,43,44^, we also used the advanced Dvorak technique (ADT) HURRSAT dataset during 1982–2009 to confirm our analyses because this is a homogeneous satellite dataset and suitable for trend analysis^45–47^.

The National Centers for Environmental Prediction/National Centers for Atmospheric Research reanalysis I (NCEP/NCAR)^48^ and the ERA-Interim reanalysis data^49^ were used to analyze the large-scale atmospheric circulation features, including 850-hPa winds and relative vorticity, sea level pressure (SLP), vertical wind shear (VWS), which was defined as the difference of vector winds between 200 and 850 hPa, and steering flow, which was defined as the mass-weighted mean winds between 300 and 850 hPa. The monthly mean SST data were derived from the Hadley Center Sea Ice and Sea Surface Temperature dataset^50^ (HadISST). We focused on the atmospheric and oceanic conditions during the typhoon season of the Northwest Pacific, namely July–November (JASON).

The atmospheric general circulation model (AGCM) ECHAM4.8 developed by the Max Planck Institute (MPI) was adopted to conduct five experiments as listed in Table 1. The ECHAM model was configured with 19 vertical levels and run at spectral T42 horizontal resolution with the mass-flux type convective parameterization scheme^51,52^. A detailed model description can be found in Roeckner *et al*.^53^. The control run (CTRL) was forced by the observed climatological monthly mean SST during 1980–2015. The hiatus run (HTS) was identical to the CTRL except that the monthly SST difference between the hiatus (1998–2015) and pre-hiatus (1980–1997) were added to each climatological monthly mean SST in the belt region (60°S–60°N; 0°–360°). To estimate the relative contributions of SST anomalies over the Pacific Ocean (PO), the IO, and the AO to the trends in atmospheric conditions controlling the ITCT change, we further conducted the PO run, the IO run, and the AO run. These three runs were also identical to the CTRL run except that the hiatus SST differences were prescribed only over the PO (60°S–60°N; 110°E–60°W), the IO (60°S–60°N; 0°–110°E), and the AO (60°S–60°N; 60°W–0°), respectively. The model was integrated for 30 years for all experiments and the last 20-year model outputs were used in our analyses.

The Singular Value Decomposition (SVD) was used to identify the coupled modes between the global SST anomalies and the ITCTs over the Northwest Pacific. The SVD was first introduced to document the simultaneous relationship between two meteorological fields^54^ and further developed to find the coupled patterns in climate variables^55^. The Empirical Orthogonal Function (EOF) analyses of the global SST anomalies and the ITCTs over the Northwest Pacific were also conducted to compare with results based on the SVD analyses. Since we focused on long-term changes, the interannual signals (less than 10 years) in the ITCT and SST data were removed using fast Fourier transform (FFT) before the SVD was performed. The Mann-Whitney U test^56^ was used to check the significance of the composite differences between hiatus and pre-hiatus periods.

### Data availability

The JTWC best track TC data are downloaded from https://metoc.ndbc.noaa.gov/web/guest/jtwc/ best_tracks/western-pacific. The SST analysis data are downloaded from the Hadley Centre website http://www.metoffice.gov.uk/hadobs/hadisst/data/download.html. The ERA-Interim data are downloaded from http://apps.ecmwf.int/datasets/. The NCEP/NCAR reanalysis I is downloaded from https://www.esrl.noaa.gov/psd/data/gridded/data.ncep.reanalysis.html.
